# Plasma Aβ42/40 ratio alone or combined with FDG-PET can accurately predict amyloid-PET positivity: a cross-sectional analysis from the AB255 Study

**DOI:** 10.1186/s13195-019-0549-1

**Published:** 2019-12-01

**Authors:** Virginia Pérez-Grijalba, Javier Arbizu, Judith Romero, Elena Prieto, Pedro Pesini, Leticia Sarasa, Fernando Guillen, Inmaculada Monleón, Itziar San-José, Pablo Martínez-Lage, Josep Munuera, Isabel Hernández, Mar Buendía, Oscar Sotolongo-Grau, Montserrat Alegret, Agustín Ruiz, Lluis Tárraga, Mercè Boada, Manuel Sarasa, The AB255 Study Group, Miguel Goñi, Francesc Pujadas, Alberto Villarejo, Ana Frank, Jordi Peña-Casanova, Manuel Fernández, Gerard Piñol, Rafael Blesa, Pedro Gil, Luis F. Pascual, Miquel Aguilar, Giovanni B. Frisoni, Jorge Matias-Guiu, Niels Andreasen, Carmen Antúnez

**Affiliations:** 1 Araclon Biotech S.L., Vía Hispanidad 21, 50009 Zaragoza, Spain; 2 Servicio de Medicina Nuclear, Clínica Universidad de Navarra, Pamplona, Spain; 3 Center for Research and Advanced Therapies and Memory Clinic, Fundación CITA-Alzheimer, San Sebastián, Spain; 4 Institut de recerca Sant Joan de Déu, Hospital Infantil Sant Joan de Déu, Barcelona, Spain; 5 Research Center and Memory Clinic, Fundació ACE, Institut Català de Neurociències Aplicades, Universitat Internacional de Catalunya-Barcelona, Barcelona, Spain; 6 Networking Research Center on Neurodegenerative Diseases (CIBERNED), Instituto de Salud Carlos III, Madrid, Spain; 7 https://www.araclon.com/alzheimer/abtest/current-situation/

**Keywords:** Plasma, Amyloid-beta, FDG-PET, Biomarker, Preclinical Alzheimer’s disease, Amyloid-PET, Mild cognitive impairment

## Abstract

**Background:**

To facilitate population screening and clinical trials of disease-modifying therapies for Alzheimer’s disease, supportive biomarker information is necessary. This study was aimed to investigate the association of plasma amyloid-beta (Aβ) levels with the presence of pathological accumulation of Aβ in the brain measured by amyloid-PET. Both plasma Aβ42/40 ratio alone or combined with an FDG-PET-based biomarker of neurodegeneration were assessed as potential AD biomarkers.

**Methods:**

We included 39 cognitively normal subjects and 20 patients with mild cognitive impairment from the AB255 Study who had undergone PiB-PET scans. Total Aβ40 and Aβ42 levels in plasma (TP42/40) were quantified using ABtest kits. Subjects were dichotomized as Aβ-PET positive or negative, and the ability of TP42/40 to detect Aβ-PET positivity was assessed by logistic regression and receiver operating characteristic analyses. Combination of plasma Aβ biomarkers and FDG-PET was further assessed as an improvement for brain amyloidosis detection and diagnosis classification.

**Results:**

Eighteen (30.5%) subjects were Aβ-PET positive. TP42/40 ratio alone identified Aβ-PET status with an area under the curve (AUC) of 0.881 (95% confidence interval [CI] = 0.779–0.982). Discriminating performance of TP42/40 to detect Aβ-PET-positive subjects yielded sensitivity and specificity values at Youden’s cutoff of 77.8% and 87.5%, respectively, with a positive predictive value of 0.732 and negative predictive value of 0.900. All these parameters improved after adjusting the model for significant covariates. Applying TP42/40 as the first screening tool in a sequential diagnostic work-up would reduce the number of Aβ-PET scans by 64%. Combination of both FDG-PET scores and plasma Aβ biomarkers was found to be the most accurate Aβ-PET predictor, with an AUC of 0.965 (95% CI = 0.913–0.100).

**Conclusions:**

Plasma TP42/40 ratio showed a relevant and significant potential as a screening tool to identify brain Aβ positivity in preclinical and prodromal stages of Alzheimer’s disease.

## Background

Alzheimer’s disease (AD) is a progressive neurodegenerative disorder and the most common cause of dementia. Accumulating data from clinical research support that the AD pathophysiologic process starts decades before the onset of clinical symptoms [1–3]. The disease develops in a continuum from a preclinical stage in which amyloid pathology has been defined as the earliest Alzheimer’s pathological changes [3–5]. Thus, individuals at the preclinical or prodromal AD stages represent an important target group in the context of clinical trials and population screening. New diagnostic procedures can identify measurable brain changes by positron emission tomography (PET) and cerebrospinal fluid (CSF) analysis, even at the preclinical stage. These procedures have been incorporated in the National Institute of Aging–Alzheimer’s Association A/T/(N) research framework for the biological definition of AD [5].

A large number of clinical studies very consistently show that these neuroimaging or CSF biomarkers provide relevant information for the diagnosis. Yet, blood-based biomarkers are the desirable tool for large-scale assessments of patients either in clinical research or, eventually, in primary clinical settings, due to their cost-effectiveness and easiness of procedures [6].

Despite previous studies yielding contradictory results [7–9], recent works have shown an association of low plasma Aβ42/40 ratio with AD [10–13]. Plasma Aβ42/40 ratio has also demonstrated value in detecting brain Aβ pathological changes [14–16], even using different methodological approaches as mass spectrometry (MS) and new-generation immunological methods [17–19]. Our group has been largely working on the development of reliable and informative plasma biomarkers based on Aβ measurements [20]. We have previously reported a consistent association between a low TP42/40 ratio and both clinical MCI diagnosis [21] and Aβ accumulation in the brain [22, 23]. Reduced TP42/40 levels were also found to predict higher rates of Aβ accumulation in the brain [22, 24]. Furthermore, it has been recently reported that lower TP42/40 ratio levels were also associated with increased cortical uptake of the [18F]Flortaucipir tau-PET marker in AD-related regions [25].

PET imaging, using the most widely available radiotracer ([18F]fluorodeoxyglucose PET, FDG-PET) as a measure of cerebral glucose metabolism, is a marker of neurodegeneration that has been established as a sensitive tool for detecting neuronal dysfunction [26]. FDG-PET diagnostic performance could be upgraded by the use of quantitative indices that have been developed to account for inter-observer variability and to support challenging evaluation [27–29]. Arbizu et al. have previously described FDG-based automated quantitative scores, such as the AD conversion score (AD-Conv score), which has proved to predict and detect AD dementia with good diagnostic performance [30].

This work was primarily aimed to investigate the potential value of the TP42/40 ratio as a screening tool for brain Aβ-PET positivity in a population of 59 CN and amnesic MCI (a-MCI) patients. This population was partially explored in a previous work in which clinical performance of TP42/40 was evaluated (considering clinical diagnosis as the gold standard), together with a general assessment of the correlation of TP42/40 with CSF and Aβ-PET biomarkers. With this study, we pursued confirming previous results from our own group in an independent AIBL population [22], focusing in the discriminating performance of TP42/40 in detecting Aβ positivity in the brain. Secondarily, we have explored the potential value added by the combination of Aβ plasma levels and AD-Conv score to improve prediction of Aβ-PET status at early disease stages. Furthermore, in the framework of the new biological concept of AD based in the A/T/(N) system [5], we also explored if combination of both the TP42/40 plasma ratio, as a β-amyloid marker (A), and AD-Conv score, reflecting neurodegeneration (N), might help in clinical assessment at early stages.

## Methods

### Participants

The AB255 Study is a multicenter longitudinal study with evaluations of the cognitive status of individuals at 0, 12, and 24 months, including 83 cognitively normal (CN) and 145 age-paired subjects with probable a-MCI [31], all over 64 years of age. The study was designed to evaluate the potential of blood-based Aβ biomarkers to detect AD. A complete description of the AB255 Study protocols and population details have been previously referred [21]. Clinical diagnosis of each participant was performed using an extensive neurological examination [32] and a battery of neuropsychological tests, including evaluation of global cognition using the Mini-Mental State Examination [33, 34] and verbal learning and memory by The Word List Learning test from the Wechsler Memory Scale-Third Edition (WMS-III) [35], delayed recall and a recognition task without list of interference [36], and the Free and Cued Selective Reminding Test (FCSRT) [37]. A subpopulation of 39 cognitively normal subjects and 20 patients with a-MCI who had undergone PiB-PET scans and fulfilled criteria for inclusion were considered for the present work. Participants were all recruited and assessed from 2010 to 2013 at *The Memory Clinic* from Fundació ACE (Barcelona, Spain) [38].

### Plasma analyses

EDTA blood was obtained after overnight fasting, and samples were immediately cooled to 2–8 °C until processed within 30 h of collection by centrifugation at 2500×*g* for 15 min at 4 °C. Plasma was appropriately aliquoted in polypropylene tubes and stored at − 80 °C until analysis, avoiding freeze-thaw cycles. ABtest40 and ABtest42 ELISA kits (Araclon Biotech Ltd., Spain) were used for the quantification of total Aβ40 and Aβ42 in plasma after proprietary treatment of the sample. The specific analytical procedures and performance characteristics of these tests are described elsewhere [20]. Samples were randomized and encoded by an external CRO to guarantee the validity of results.

### PiB-PET analysis

Cortical Aβ burden was assessed by PET using 11C-Pittsburg compound B (PiB-PET). Detailed procedures of neuroimaging acquisition and analysis are described by Espinosa et al. [31]. Imaging data were analyzed using the Fundació ACE Pipeline for Neuroimaging Analysis, available at http://detritus.fundacioace.com/. Participants were classified as β-amyloid positive (PET-Aβ(+)) or β-amyloid negative (PET-Aβ(−)) with relation to a cutoff of 1.4 SUVR in PiB-PET scans [39].

### FDG-PET analysis

PET acquisition was performed after 4 h of fasting and once it was confirmed that blood glucose levels were below 110 mg/dl. The dose of ^18^FDG was established according to weight (150 μCi/kg), being the standard adult dose 10 mCi (370 MBq) in a volume of 1–10 ml saline. Dosimetry was established in accordance with ICRP 53. For adults, critical organ dose (bladder) was 0.16 mGy/MBq and effective dose was 0.019 mSv/MBq. Acquisition started 40 min after ^18^FDG administration and lasted for 20 min. FDG-PET scans were analyzed following the procedure previously described by Arbizu et al. [30]. Using this automated voxel-based analytical method, quantitative indices were developed to compute the hypometabolic pattern of each subject. The AD-Conv score integrates the information provided by PET into a multivariate model including age, gender, Mini-Mental State Examination (MMSE) score, and APOE ε4 genotype.

### APOE genotyping

Genomic DNA was isolated from EDTA blood. Target DNA was amplified by PCR and digested with the restriction enzyme HhaI. APOE genotyping was carried out by subsequent restriction analysis of the pattern of fragments obtained after electrophoresis in a polyacrylamide gel [40].

### Statistical analysis

Statistical analysis was performed using SPSS version 22 for Windows (SPSS Inc., Chicago, IL). A probability level of *p* < 0.05 was considered statistically significant. Total in plasma Aβ42/40 ratio (TP42/40) and AD-Conv score were used as the predictive variables. Demographic characteristics and biomarker levels were first compared between groups using chi-squared and Mann-Whitney tests as appropriate. Statistically significant differences in TP42/40 levels between Aβ-PET(+) and Aβ-PET(−) individuals were evaluated using generalized linear regression models (GLM) adjusted for significant demographic covariates (age, APOE genotype, and clinical group). Correlation of quantitative measures of both plasma TP42/40 ratio and AD-Conv score with Aβ-PET SUVR was assessed by Spearman rank correlation analyses.

The association of TP42/40 and AD-Conv score with PET-based abnormal amyloid status was further investigated using logistic regression followed by receiver operating characteristic (ROC) curve analyses. Predicted values of binary logistic regression models were used to combine variables in ROC analysis. All TP42/40 models were adjusted for age, APOE genotype (APOEε4 allele carriers versus non-carriers), and clinical diagnosis. AD-Conv score was determined by a multivariate model already including age and APOE ε4 genotype, so it was not readjusted for these covariates in the analysis. Plasma and FDG-PET biomarkers were evaluated alone or in combination, so that improvement in classification performance using both amyloid and neurodegeneration information derived from them was assessed. Youden’s index maximizing cutoffs were considered for evaluation of biomarker performance in detecting brain Aβ positivity, either individually or combined. This same procedure was applied to the total AB255 population to assess TP42/40 and AD-Conv score clinical discrimination ability (CN versus a-MCI). For visualization purposes, heat maps representing predicted probability of brain Aβ positivity depending on the chosen TP42/40 cutoff were depicted with regard to age and APOE genotype.

## Results

**Fig. 1:**
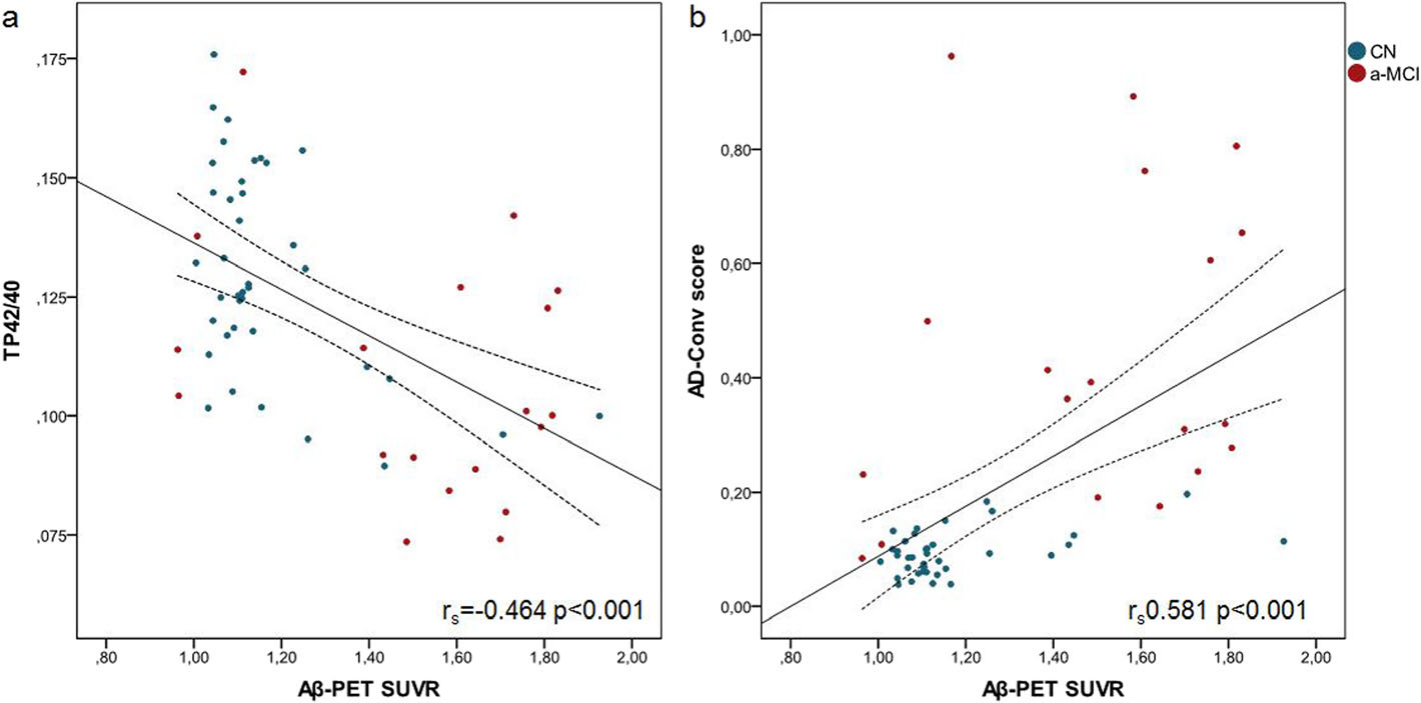
Correlation of TP42/40 and AD-Conv score with Aβ-PET. Scatterplots of plasma TP42/40 ratio (**a**) and AD-Conv score (**b**) levels with regard to PiB-PET. Blue: cognitively normal (CN) subjects. Red: amnestic mild cognitive impairment (a-MCI) individuals. Both TP42/40 and AD-Conv score biomarkers showed a significant correlation with Aβ-PET, although in inverse directions. *r*_s_ Spearman rank correlation coefficient

Demographic characteristics of the population included in this study are summarized in Table 1. Eighteen (30.5%) individuals were classified as Aβ-PET(+), and most of them were clinically diagnosed as a-MCI (14 a-MCI Aβ-PET(+) versus 6 a-MCI Aβ-PET(−)). Likewise, 35 out of the 39 CN individuals were classified as Aβ-PET(−). Comparing Aβ-PET(+) and Aβ-PET(−) groups, subjects with abnormal Aβ-PET status were on average older and were more frequently APOE Ɛ4 carriers. Age, APOE Ɛ4, and clinical diagnosis were significantly different between groups, whereas no statistically significant differences were found with regard to gender or education level (Table 1). Both AD-Conv score and TP42/40 were significantly different between Aβ-PET groups (Mann-Whitney *p* < 0.001) and correlated with Aβ-PET SUVR measures (TP42/40 *r*_s_ = − 0.464; AD-Conv score *r*_s_ = 0.581; both *p* < 0.001). Figure 1 shows these associations including information of the clinical status of each participant.

**Table 1:** Demographic and biomarker features of this population

	Aβ-PET (−)	Aβ-PET (+)	*p* value
*N* (%)	41 (69.5%)	18 (30.5%)	
Age, mean (SD)	71.6 (4.11)	75.2 (5.65)	*p* 0.014
Gender Female, *n* (%)	18 (43.9%)	9 (50.0%)	*p* 0.665
APOE4 carriers, *n* (%)	6 (14.6%)	12 (66.7%)	*p* < 0.001
Education level, mean years (SD)	12.33 (3.96)	10.56 (4.44)	*p* 0.113
CN/a-MCI, *n* (% a-MCI)	35/6 (14.6%)	4/14 (77.8%)	*p* < 0.001
TP42/40, mean (SD)	0.1329 (0.0208)	0.0997 (0.0197)	*p* < 0.001
AD-Conv score, mean (SD)	0.133 (0.162)	0.384 (0.259)	*p* < 0.001

*p* value was obtained from Mann-Whitney or chi-squared tests as appropriate. *SD* standard deviation, *CN* cognitively normal, *a-MCI* amnestic mild cognitive impairment, *Aβ* amyloid-beta, *TP42/40* total plasma Aβ42/40 ratio, *AD-Conv score* AD conversion score based on FDG-PET

**Fig. 2:**
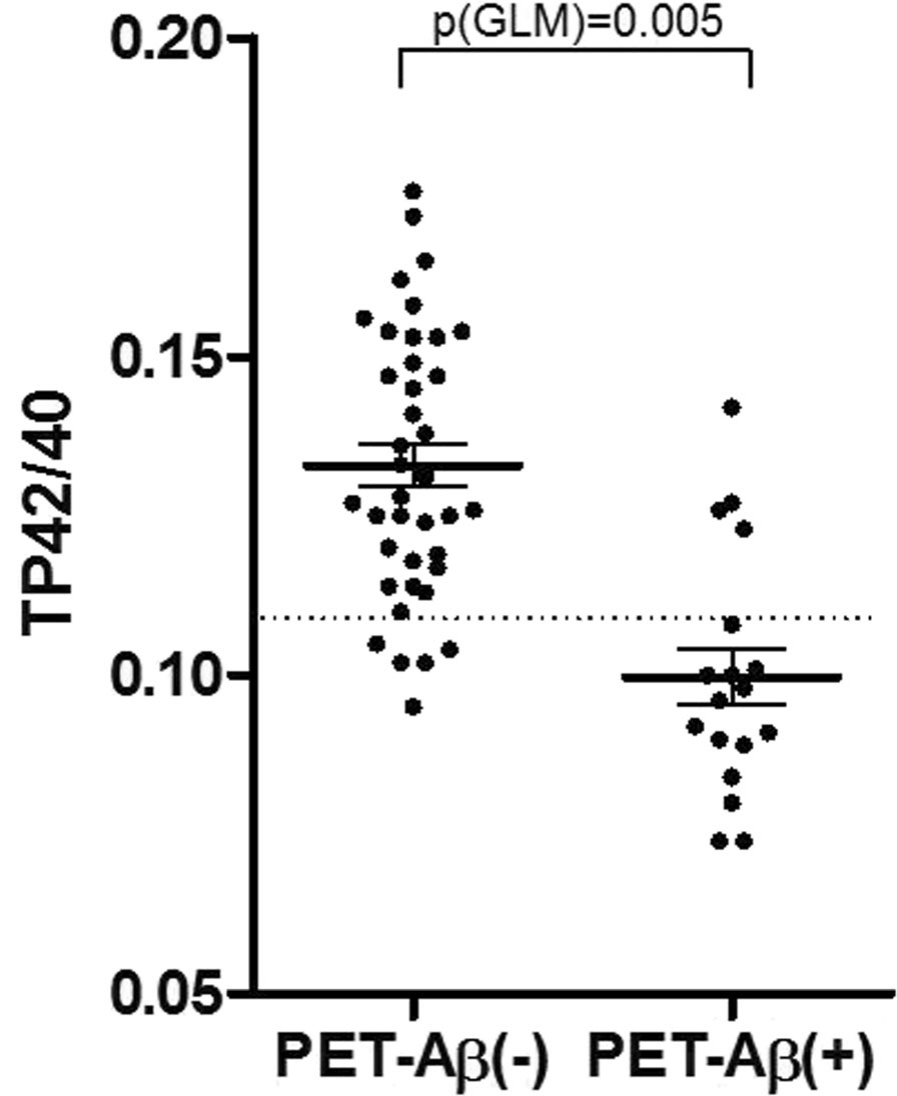
Distribution of TP42/40 levels between Aβ-PET groups. Dots represent the individual TP42/40 ratio obtained for each patient with regard to their Aβ-PET status. Continuous lines represent mean TP42/40 values and standard mean error. The dashed line represents the Youden’s cutoff (0.1094) PET-Aβ positivity discrimination using TP42/40 alone. *p* value from the generalized linear model (GLM) was also included

**Fig. 3:**
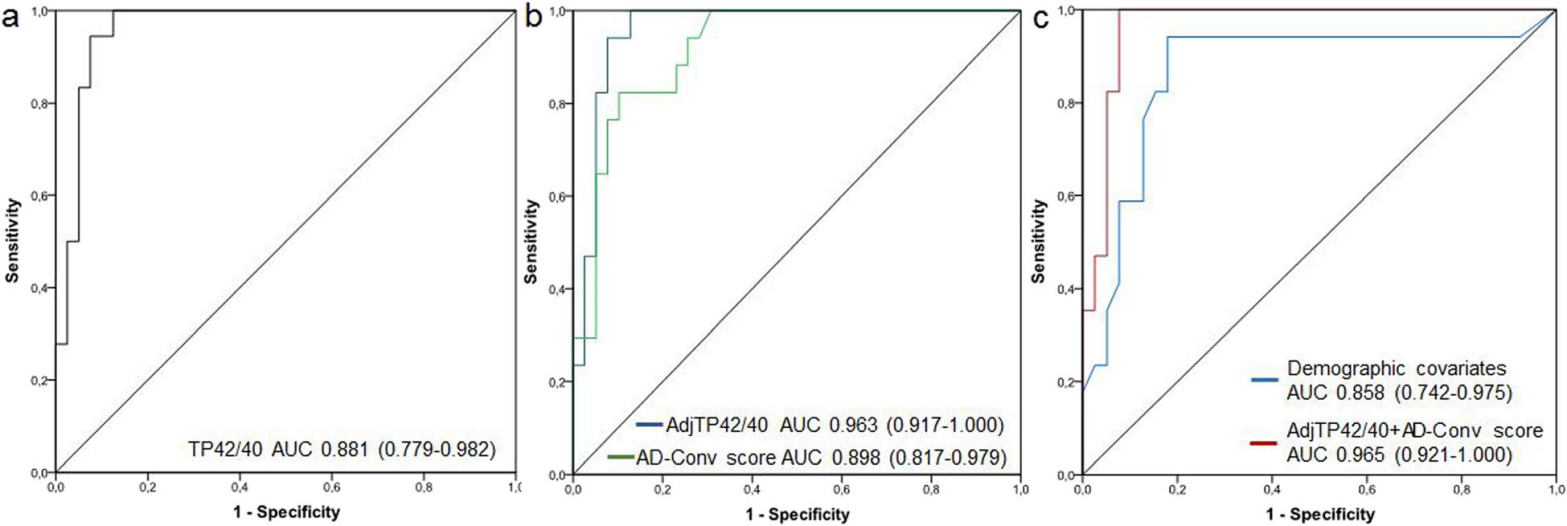
Receiver operating characteristic (ROC) curves discriminating Aβ-PET(+) from Aβ-PET(−) subjects. ROC curve and the corresponding area under the curve (AUC) with 95% confidence interval (CI) are depicted for the following models: **a** TP42/40 alone; **b** TP42/40 adjusted for age, APOE genotype, and clinical group (AdjTP42/40) and AD-Conv score; **c** models including only significant demographic covariates (age, APOE genotype, and clinical group) and the combined model of amyloidosis and neurodegeneration (AdjTP42/40+AD-Conv score)

Plasma TP42/40 ratio was in average 25% lower in PET-Aβ(+) subjects compared to PET-Aβ(−) individuals (Table 1 and Fig. 2). After adjusting for significant demographic covariates (age, APOE Ɛ4, and clinical diagnosis), association of TP42/40 with Aβ-PET status remained statistically significant (GLM *β* = 87.67, *p* = 0.005). ROC analysis revealed an AUC of 0.881 (95% confidence interval [CI] 0.779–0.982) for the prediction of Aβ-PET positivity using TP42/40 alone (Fig. 3a). The Youden’s cutoff of plasma TP42/40 ratio was 0.1049 and yielded a sensitivity of 77.8% and a specificity of 87.5% in detecting PET-Aβ(+) subjects (Table 2). When studying independently CN and a-MCI subjects, we found excellent performance of TP42/40 in the CN group (AUC unadjusted TP42/40 0.957, 95% CI 0.890–1.000), although it declined in the a-MCI group (AUC unadjusted TP42/40 0.814, 95% CI 0.617–1.000) (Table 2).

**Table 2:** Diagnostic performance of individual and combined models in detecting Aβ-PET positivity at Youden’s cutoff

	Sensitivity	Specificity	PPV	NPV	LR+	LR−
Whole population (*N* = 59)						
TP42/40	0.778	0.875	0.732	0.900	6.22	0.25
Age+APOE4+Clinical Group	0.944	0.805	0.680	0.945	4.84	0.07
TP42/40+Age+APOE4+Clinical Group	0.944	0.925	0.847	0.974	12.59	0.06
AD-Conv score	1.000	0.675	0.706	1.000	3.08	0.00
AdjustedTP42/40+AD-Conv score	1.000	0.923	0.851	1.000	13.00	0.00
Only CN (*N* = 39)						
TP42/40	1.000	0.886	0.500	1.000	8.75	0.00
Age+APOE4	0.750	0.943	0.600	0.971	12.12	0.26
TP42/40+Age+APOE4	1.000	1.000	1.000	1.000	–	0.00
AD-Conv score	1.000	0.765	0.327	1.000	4.25	0.00
AdjustedTP42/40+AD-Conv score	1.000	1.000	1.000	1.000	–	0.00
Only a-MCI (*N* = 20)						
TP42/40	0.714	1.000	1.000	0.653	–	0.29
Age+APOE4	0.357	0.833	0.799	0.411	2.14	0.77
TP42/40+Age+APOE4	0.643	1.000	1.000	0.601	–	0.36
AD-Conv score	0.846	0.500	0.759	0.636	1.70	0.31
AdjustedTP42/40+AD-Conv score	0.615	1.000	1.000	0.583	–	0.38

Cases in which LR+ was not quantifiable were presented as –. Prevalence considered for each group calculation was corresponding to the present cohort: 30.5% in the whole population, 10.25% in the CN group, and 65% in the a-MCI group. *PET PPV*-positive predictive value, *NPV* negative predictive value, *LR+* positive likelihood ratio, *LR−* negative likelihood ratio

**Fig. 4:**
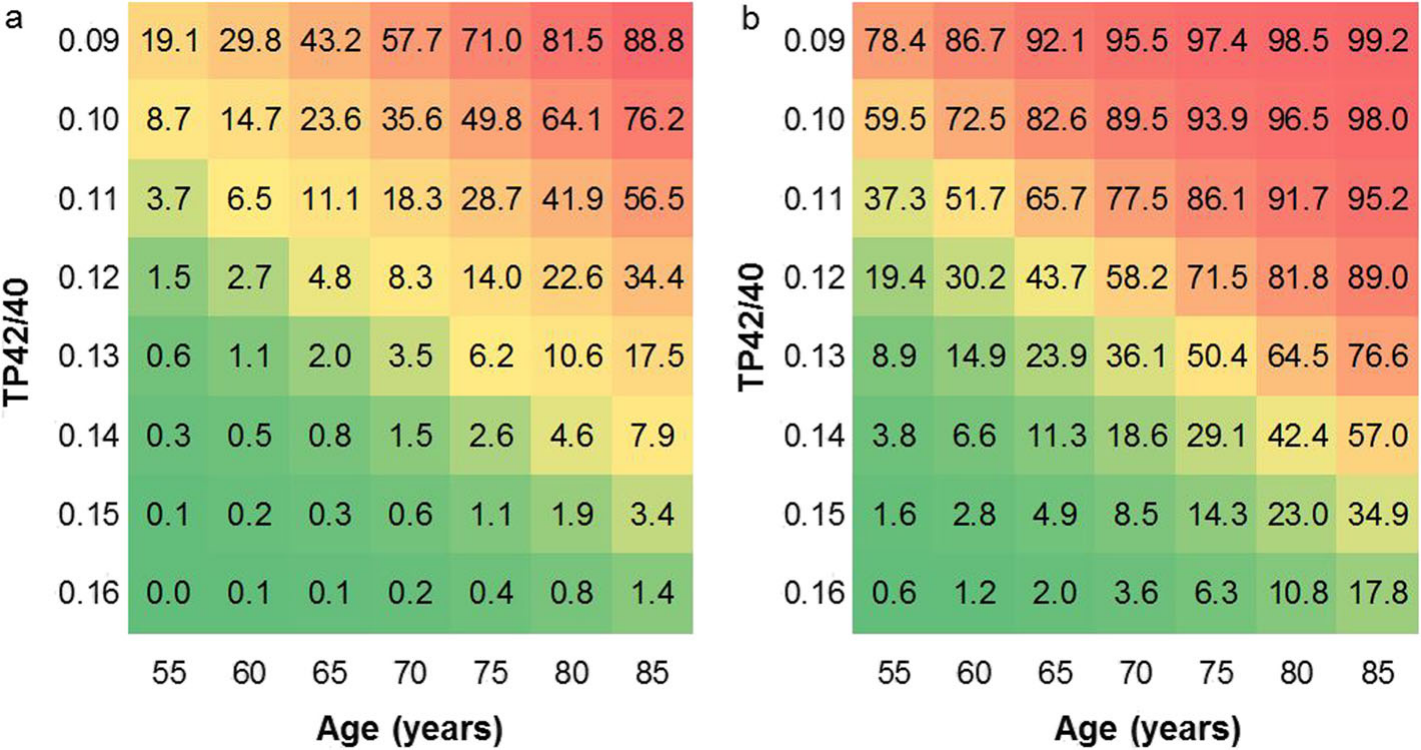
Heat maps showing predicted probability of being Aβ-PET(+) based on TP42/40 and age stratified for APOE genotype. Predicted probabilities in APOE Ɛ4 non-carriers (**a**) and APOE Ɛ4 carriers (**b**). Probabilities are displayed as percentages. Note that TP42/40 decreases upward in axis for optimal visualization purposes. It should be noted that probabilities were predicted from a logistic regression model modeling expected probabilities in the age range most likely present during pre-screening or clinical assessment. These predicted probabilities could be more accurately determined in a larger population including the complete age range

When adjusting TP42/40 with relevant covariates (age, APOE genotype, and clinical group), discrimination was improved yielding an AUC of 0.963 (95% CI 0.917–1.000). Sensitivity, specificity, and NPV were all over 0.920 (Fig. 3b, Table 2). We used the logistic regression model to predict probabilities of Aβ-PET positivity with regard to age in both APOE ε4 non-carriers (Fig. 4a) and carriers (Fig. 4b). This figure represents a statistical inference since the actual study population does not cover all the age ranges represented. As expected, the predicted probability increased with age and was always higher in APOE ε4 carriers compared to non-carriers. Low TP42/40 ratio considerably augmented likelihood of abnormal Aβ-PET status for every genotype and age condition. Indeed, the high NPV and low LR− characterized TP42/40 as an ideal marker for pre-screening. Considering a hypothetical clinical trial targeting Aβ-PET-positive subjects, a recruitment procedure based in Aβ-PET alone would require 3.3 PET scans per successful recruitment. However, following a sequential strategy including a TP42/40 pre-screening stage would require 1.2 Aβ-PET scans per successful recruitment, implying a 64% saving in PET scans.

AD-Conv score was also evaluated as a predictor of Aβ-PET positivity in this cohort, showing good diagnostic performance (AUC 0.898, 95% CI 0.817–0.979) (Fig. 3b), and sensitivity and specificity values at maximum Youden’s cutoff of 100% and 67.5%, respectively (Table 2). Thus, we hypothesized that a combination of both plasma TP42/40 and neurodegeneration (FDG-PET) biomarkers could outperform the single-predictor models in detecting Aβ-PET(+) subjects.

The combined model of plasma Aβ and neurodegeneration biomarkers (AdjTP42/40+AD-Conv score) improved performance of both individual biomarkers and covariates alone (Fig. 3c) to discriminate Aβ-PET status. AUC for this model was found to be 0.965 (95% CI 0.921–1.000), with very high sensitivity and specificity (> 92%) and NPV of 1.00 (Table 2).

On the other hand, TP42/40 performed poorly at discriminating between CN and a-MCI patients in the whole AB255 study population (AUC = 0.639, 95% CI 0.564–0.714). Nevertheless, combining TP42/40 with AD-Conv score improved subjects’ clinical classification up to an AUC of 0.915 (95% CI 0.875–0.955). The effect is mainly driven by AD-Con score which by itself alone presented an AUC of 0.893 (95% CI 0.849–0.937).

## Discussion

In this study, we aimed at assessing the potential value of TP42/40 ratio in the prediction of brain Aβ pathology measured by Aβ-PET in a preclinical and prodromal AD cohort of 59 patients from the AB255 Study. In addition, the present study evaluated the potential benefit of the combination of amyloid and neurodegeneration biomarkers, reflected by plasma TP42/40 and the FDG-PET-derived AD-Conv score [30]. Both individually and combined, these biomarkers significantly detected Aβ pathology in the brain. Combination of adjusted TP42/40 ratio and AD-Conv score was found to be the most accurate Aβ-PET predictor, with sensitivity of 100% and specificity of 92.3%.

Aβ-PET(+) patients showed significantly lower levels of TP42/40, with an average reduction of 25% compared to the Aβ-PET(−) group. This significant inverse association of plasma Aβ42/40 ratio with brain Aβ deposition is in accordance with previous studies [14–19]. These findings also replicate our previous results showing the potential of TP42/40 as a surrogate biomarker of Aβ-PET status in an independent preclinical population [22]. TP42/40 ratio adjusted for significant covariates detected Aβ-PET positivity in the present cohort with 94.4% sensitivity and 92.5% specificity. This means that in a sequential screening scenario aiming to detect subjects with abnormal Aβ-PET status, a first step assessing TP42/40 would allow a 64% reduction in the number of Aβ-PET scans. Considering the restricted availability and costs of Aβ-PET neuroimaging [41], this sequential strategy would imply a significant reduction of the patient burden and procedural costs, both for clinical trials and patient management at primary care settings.

Clinical performance of plasma Aβ42/Aβ40 in the present study is comparable to that obtained using MS-based methods [17, 18] or new-generation immunological assays [19] and are congruent with other studies demonstrating a consistent inverse association of the plasma Aβ42/Aβ40 with the disease [10–13, 42, 43]. Nevertheless, there still exists considerable overlap of plasma Aβ ratio between diagnostic groups or amyloid status groups, which limits the use of Aβ plasma ratios for clinical diagnosis at the individual level. On the other hand, clinical performance of plasma biomarkers is also hampered by the fact that Aβ-PET classification, used as the gold standard, neither is utterly precise. In line with this, plasma TP42/40 false-positive results could correspond to a-MCI subjects who are already accumulating Aβ in the brain, but have not reached the threshold for abnormal status yet. If this were the case, plasma Aβ biomarkers would turn out to be abnormal before Aβ-PET does. Alternatively, misclassification could be due to heterogeneity of the underlying disease in a-MCI patients. When using only CN to explore TP42/40 potential in detecting Aβ-PET+ subjects, results are better compared to the a-MCI group or the mixed population. Adjusted TP42/40 can accurately detect the 4 AB-PET+ individuals in the cohort of 39 CN, which confirms the preclinical potential of this biomarker when detecting Aβ abnormality in the brain, detected also in previous work from our group [22]. However, due to the small size of the CN and a-MCI groups in the present population, together with the different prevalence of Aβ-PET positivity, these findings should be taken cautiously and further replicated.

AD-Conv score has proven a significant correlation with Aβ-PET and a good performance in detecting brain Aβ pathology within this cohort, being the AUC values obtained within the range previously defined [30]. These results reinforce the suitability of quantitative indices derived from automated FDG-PET measures replacing standard qualitative brain FDG-PET imaging [27–29].

Previous studies evaluating the association of FDG-PET and Aβ-PET measures have shown discrepant results. Several works did not find a correlation in AD [44–46], MCI [47], and CN individuals [48, 49], although it was found in other cohorts [50–54]. Another study including both CN and MCI individuals showed a significant correlation between PiB-PET and FDG-PET [55], corresponding well to our findings.

Only few studies have explored combination of FDG-PET and Aβ biomarkers, mostly in CSF and strictly focused on conversion to AD dementia [43, 46–48]. Interestingly, combination of TP42/40 and AD-Conv score improves subjects’ clinical classification into CN or a-MCI. As could be expected, the effect is mainly driven by the AD-Conv score which itself integrates into a multivariate model FDG-PET neurodegeneration (downstream to β-amyloid changes as per the amyloid cascade hypothesis) with APOE genotype, MMSE score, age, and gender. This approach is in agreement with the recent research framework based on the A/T/(N) system [5], and our findings support the suitability of a biological definition of AD, measured by biomarkers that are reflecting different processes underlying AD clinical manifestations.

Larger and varied populations need to be explored in order to confirm the potential utility of FDG-PET and TP42/40 combination, which may vary depending on population characteristics and disease stage. In fact, the overall improvement of the adjusted TP42/40 model by AD-Conv score, although relevant, was relatively modest in our cohort. Another weakness of our study is the absence of a PiB-PET follow-up, which would be of great utility in ascertaining the potential prognostic value of plasma biomarkers to predict the rate of brain Aβ deposition and could have provided relevant evidence for elucidating the possible source of bias in the classification performance.

## Conclusions

This study demonstrates a consistent inverse association of plasma TP42/40 and FDG-PET biomarkers with Aβ-PET status. Findings from this work replicate our previous results in different cohorts, hence confirming TP42/40 as a useful biomarker to rule out brain β-amyloidosis in preclinical and early AD stages. Plasma TP42/40 tests may be used as an initial screening tool for patient management; only those resulting TP42/40 positives would have to undergo diagnostic confirmation by CSF analysis or PET neuroimaging. Integration of neurodegeneration and blood-based Aβ biomarkers could contribute to early AD diagnosis in compliance with the A/T/(N) research framework, although it requires further validation.

## Data Availability

The datasets used and/or analyzed during the current study are available from the corresponding author on reasonable request.

## Abbreviations

AD: Alzheimer’s disease
PET: Positron emission tomography
CSF: Cerebrospinal fluid
CN: Cognitively normal
MCI: Mild cognitive impairment
Aβ-PET: Amyloid-beta PET
MS: Mass spectrometry
Aβ: Amyloid-beta
TP42/40: Total in plasma Aβ42/40
AIBL: Australian Imaging Biomarkers and Lifestyle Study of Aging
FDG-PET: [18F]fluorodeoxyglucose PET
AD-Conv score: AD conversion score (FDG-PET)
a-MCI: Amnestic MCI
CRO: Contract research organization
ROC: Receiver operating characteristic
CI: Confidence interval
GLM: Generalized linear regression models
PPV: Positive predictive value
NPV: Negative predictive value
LR: Likelihood ratio
